# Low bone mineral density in chronic hepatitis B virus infection: A case-control study

**DOI:** 10.12669/pjms.332.12099

**Published:** 2017

**Authors:** Zengfa Huang, Hui Wei, Cheng Cheng, Shuhua Yang, Jing Wang, Xianzhe Liu

**Affiliations:** 1 Zengfa Huang, Department of Orthopedics, Union Hospital, Tongji Medical College, Huazhong University of Science and Technology, Wuhan, China; 2 Hui Wei, Department of Orthopedics, Union Hospital, Tongji Medical College, Huazhong University of Science and Technology, Wuhan, China; 3 Cheng Cheng, Department of Orthopedics, Union Hospital, Tongji Medical College, Huazhong University of Science and Technology, Wuhan, China; 4 Shuhua Yang, Department of Orthopedics, Union Hospital, Tongji Medical College, Huazhong University of Science and Technology, Wuhan, China; 5 Jing Wang, Department of Orthopedics, Union Hospital, Tongji Medical College, Huazhong University of Science and Technology, Wuhan, China; 6 Xianzhe Liu, Department of Orthopedics, Union Hospital, Tongji Medical College, Huazhong University of Science and Technology, Wuhan, China

**Keywords:** BMD, Chronic HBV infection, Chronic liver disease, Osteoporosis, Osteopenia

## Abstract

**Objective:**

Osteoporosis is the well-known major complication in chronic hepatitis B virus (HBV) infection. Fewer reports are available of the relationship between bone loss and chronic HBV infection. We investigated the bone mineral density (BMD) and prevalence of osteoporosis in chronic HBV patients in comparison with healthy subjects.

**Methods:**

We assessed 148 chronic HBV patients and 148 age- and gender-matched healthy controls by dual energy X-ray absorptiometry (DEXA) for determination of BMD. T-score was used to define bone status according to the World Health Organization’s classification.

**Results:**

The BMD values were significantly lower in HBV patients in all scan of specific regions compared with the controls (*P* < 0.05). The prevalence of osteoporosis in either of lumbar spine (LS), total hip (TH) or the femoral neck (FN) was significantly higher in the HBV patients group compared with the healthy controls. The rate of osteopenia and osteoporosis for HBV patients aged 45–54 years was significantly higher than that of the healthy controls.

**Conclusions:**

Chronic HBV infection was associated with low BMD and increased the risk of developing subsequent osteoporosis.

## INTRODUCTION

Osteoporosis is an escalating disease with high morbidity and mortality rates and large socioeconomic expenses. The term “hepatic osteodystrophy” is a common complication among chronic liver disease (CLD) patients.^1^ It is a metabolic bone disease secondary to increased resorption and reduced formation of bone.^2^ Osteoporosis is the well-known major complication in this chronic hepatitis.^3^ The prevalence of osteoporosis in CLD varies widely, ranges from 20 to 50% depending on patient selection and diagnostic criteria.^4^ Many researches have reported significant osteoporosis in patients with cirrhosis, especially secondary to hepatitis B.^1^,^2^

Hepatitis B is a global health problem caused by infection with the hepatitis B virus (HBV); it is estimated that as many as 350 million people are affected with HBV worldwide, and about 75% of them are from Asia.^5^ In addition, many extrahepatic manifestations are associated with chronic HBV infection.^6^ However, fewer reports are available of the relationship between bone loss and chronic HBV infection.

To determine whether chronic HBV infection is at increased risk of developing low bone mass, we investigated the prevalence of osteoporosis in chronic HBV patients in comparison with age- and sex-matched healthy subjects.

## METHODS

A total of 148 patients with chronic HBV patients were assessed, they were recruited at Department of Infectious Diseases, Union Hospital, Tongji Medical College from January 2014 to December 2015 and were defined by clinical, biochemical, serological, immunological and histopathological investigations with duration up to 6 months. Also, we selected 148 healthy controls with age- and gender-matched from the Medical Examination Center, Union Hospital, Tongji Medical College. The study protocol was approved by the Ethics Committee of Union Hospital, Tongji Medical College, according to the ethical and moral principles stated in the Helsinki 1966 Declaration on Human Rights.

Demographics, lifestyle, and menopause status of study participants were collected. Exclusion criteria were: any treatments that could affect bone mass (calcium, bisphosphonates, estrogens, vitamin D supplements, corticosteroids, and active intravenous drugs.), history of hip or lumbar spine fracture, history of malignancy, severe heart disease, chronic kidney disease, human immunodeficiency virus (HIV) coinfection, malnutrition, and other chronic liver disease, such as chronic hepatitis C, primary biliary cirrhosis, alcoholic liver disease, autoimmune liver disease, bone disorders.

### Bone mineral density measurements

Bone mineral density of lumbar spine (LS, L1–L4), total hip (TH), femoral neck (FN), trochanter (Tro) and ward’s triangle (WT) were measured by dual-energy X-ray absorptiometry (DXA) (QDR-4500A; Hologic, Waltham, MA). All measurements were realized by two technicians in all patients and controls. According to the World Health Organization criteria,^7^ osteoporosis was defined as a T score below -2.5 SD, osteopenia was defined as a T score between -1 and -2.5 SD and low BMD was defined as Z score below -2.

### Statistical Analysis

All data were expressed as mean ± SD. The Chi square test was used to analyze differences in non-continuous variables and the Student’s t test in continuous variables. *P* < 0.05 was considered as a significant difference. All statistical analyses were performed by SPSS version 13 (SPSS, Inc., Chicago, IL).

## RESULTS

The demographic and clinical characteristics of the HBV patients and the healthy controls are presented in Table-I. The mean age of HBV patients was 43.7 ± 11.9 years (range 15–72 years). There were 26 (17.8%) females and 122 (82.4%) males. The mean BMI was 22.7 ± 1.9 kg/m^2^ (range 17.7–27.7). A total of 42.3% (11/26) of the females in HBV patients were postmenopausal.

**Table-I:** The demographic and clinical characteristics of the patients and the controls.

	*HBV Patients*	*Healthy Controls*	*P*value
N	148	148	
Sex (M, %)	122(82.4)	122(82.4)	
Age (years, Mean ± SD)	43.7(11.9)	43.6(11.9)	0.581
BMI (kg/m^2^, Mean ± SD)	22.7(1.9)	23.0(1.8)	0.925
Smoke (n, %)	91(61.5)	87(58.8)	0.225
Menopause (n, %)	11(42.3)	11(42.3)	

M = male, BMI = body mass index.

BMD values (g/cm^2^), Z scores and T scores of the lumbar spine, femoral neck, total hip, trochanter and ward’s triangle in HBV patients and healthy controls are shown in Table-II. The BMD values were significantly lower in HBV patients in all scan of specific regions compared with the controls (*P* < 0.05). In addition, the T scores and Z scores showed significantly lower than the controls at the lumbar spine, femoral neck and total hip sites except Z scores in women.

**Table-II:** Comparison of BMD, T and Z-scores between HBV patients and healthy controls.

*All*			*Men*			*Women*		
	*HBV patients (Mean ± SD)*	*Healthy Controls (Mean ± SD)*	*P value*	*HBV patients (Mean ± SD)*	*Healthy Controls (Mean ± SD)*	*P value*	*HBV patients (Mean ± SD)*	*Healthy Controls (Mean ± SD)*	*P value*
N	148	148		122	122		26	26	
BMD, g/cm^2^									
TH	0.874 ± 0.058	0.945 ± 0.060	0.01	0.871 ± 0.047	0.952 ± 0.047	0.01	0.860 ± 0.093	0.910 ± 0.093	0.01
FN	0.829 ± 0.060	0.915 ± 0.061	0.01	0.825 ± 0.052	0.921 ± 0.050	0.01	0.823 ± 0.093	0.889 ± 0.094	0.01
Tro	0.732 ± 0.067	0.797 ± 0.070	0.01	0.748 ± 0.045	0.819 ± 0.043	0.01	0.642 ± 0.078	0.694 ± 0.078	0.01
WT	0.756 ± 0.075	0.823 ± 0.076	0.01	0.771 ± 0.059	0.844 ± 0.055	0.01	0.658 ± 0.082	0.724 ± 0.081	0.01
T-score									
LS	-1.05 ± 0.87	-0.49± 0.76	0.01	-1.15 ± 0.87	-0.32 ± 0.64	0.01	-1.23 ± 0.85	-0.56 ± 0.74	0.080
TH	-1.05 ± 0.88	-0.52 ± 0.62	0.01	-1.16 ± 0.85	-0.47 ± 0.57	0.01	-0.65 ± 0.84	-0.56 ± 0.81	0.648
FN	-1.12 ± 0.91	-0.50 ± 0.60	0.01	-1.26 ± 0.88	-0.49 ± 0.52	0.01	-0.55 ± 0.90	-0.51 ± 0.74	0.851
Z-score									
LS	-0.42 ± 0.56	-0.04 ± 0.43	0.01	-0.39 ± 0.56	0.03 ± 0.38	0.01	-0.56 ± 0.54	-0.42 ± 0.46	0.361
TH	-0.38 ± 0.57	0.16 ± 0.46	0.01	-0.47 ± 0.53	0.20 ± 0.39	0.01	-0.04 ± 0.55	0.04 ± 0.66	0.613
FN	-0.44 ± 0.62	-0.10 ± 0.40	0.01	-0.55 ± 0.56	-0.14 ± 0.37	0.01	0.07 ± 0.64	0.04 ± 0.50	0.850

BMD = bone mineral density, LS = lumbar spine, TH = total hip, FN = femoral neck, Tro = trochanter, WT = ward’s triangle.

**Fig.1:**
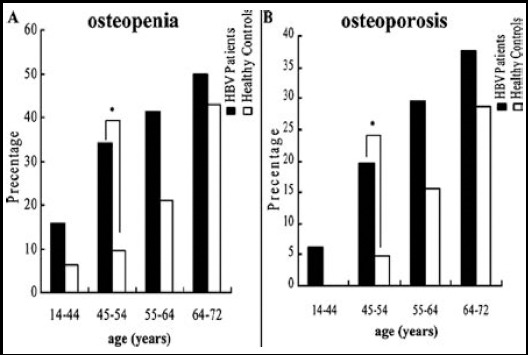
Comparison of the prevalence of osteopenia (A), osteoporosis (B) between chronic HBV patients and health controls. *P < 0.05.

The prevalence of osteopenia was showed in Table-III. The prevalence of osteopenia in HBV patients at LS, TH and FN were 23.6%, 24.3%, 23%, respectively. In healthy control, 10.1%, 9.5%, 9.5% participants were osteopenic at LS, TH and FN, respectively. The prevalence of osteopenia was significantly higher in male and female HBV patients regardless of their menopausal status than their controls. The prevalence of osteoporosis is shown in Table-IV. The prevalence of osteoporosis in either of LS, TH or the FN was significantly higher in the HBV patients group (19/148, 12.8%; 17/148, 11.5%; 18/148, 12.2%) compared with the healthy control (7/148, 4.7%; 6/148, 4.1%; 7/148, 4.7%) (*P* = 0.022; *P* = 0.028; *P* = 0.035). When the prevalence of osteopenia and osteoporosis were evaluated according to ranges of age, all categories showed higher rates of osteopenia and osteoporosis in HBV patients than the healthy controls with a statistically significant difference for HBV patients between 45 and 54 years (*P* = 0.008, *P* = 0.048) (Fig.1).

**Table-III:** Prevalence of osteopenia in chronic HBV patients and healthy controls.

	*LS*			*TH*			*FN*		

	*HBV Patients*	*Healthy Controls*	**P*value*	*HBV Patients*	*Healthy Controls*	*P value*	*HBV Patients*	*Healthy Controls*	*P value*
All	35/148(23.6%)	15/148(10.1%)	0.003	36/148(24.3%)	14/148(9.5%)	0.001	34/148(23%)	14/148(9.5%)	0.002
Men	22/122(18%)	10/122(8.2%)	0.036	22/122(18%)	9/122(7.4%)	0.02	21/122(17.2%)	9/122(7.4%)	0.031
Women	13/26(50%)	5/26(19.2%)	0.04	14/26(53.8%)	5/26(19.2%)	0.02	13/26(50%)	5/26(19.2%)	0.04
Menopausal women	5/12(41.7%)	3/12(25%)	0.667	6/12(50%)	3/12(25%)	0.4	5/12(41.7%)	3/12(25%)	0.667
Non-menopausal women	8/14(57.1%)	2/14(14.3%)	0.046	8/14(61.5%)	2/14(14.3%)	0.046	8/14(57.1%)	2/14(14.3%)	0.046

LS = lumbar spine, TH = total hip, FN = femoral neck.

**Table-IV:** Prevalence of osteoporosis in chronic HBV patients and healthy controls.

	*LS*			*TH*			*FN*		

	*HBV Patients*	*Healthy Controls*	*P value*	*HBV Patients*	*Healthy Controls*	*P value*	*HBV Patients*	*Healthy Controls*	*P value*
All	19/148(12.8%)	7/148(4.7%)	0.022	17/148(11.5%)	6/148(4.1%)	0.028	18/148(12.2%)	7/148(4.7%)	0.035
Men	11/122(9%)	4/122(3.3%)	0.107	10/122(8.2%)	3/122(2.5%)	0.084	10/122(8.2%)	4/122(3.3%)	0.167
Women	8/26(30.7%)	3/26(11.5%)	0.173	7/26(26.9%)	3/26(11.5%)	0.291	8/26(30.7%)	3/26(11.5%)	0.173
Menopausal women	4/12(33.3%)	2/12(16.7%)	0.64	3/12(25%)	2/12(16.7%)	0.615	4/12(33.3%)	2/12(16.7%)	0.64
Non-menopausal women	4/14(28.5%)	1/14(7.1%)	0.326	4/14(28.5%)	1/14(7.1%)	0.326	4/14(28.5%)	1/14(7.1%)	0.326

LS = lumbar spine, TH = total hip, FN = femoral neck.

## DISSCUSSION

The present study reveals a higher prevalence of osteoporosis and osteopenia in HBV patients than healthy controls. However, the overall prevalence of osteoporosis in previous studies with chronic liver diseases patients was ranging from 12% to 55% and that prevalence in viral hepatitis patients was 20% to 55%.^2^ The total prevalence of osteoporosis in the present study was 12.8% in HBV patients.

Our study showed that the rate of osteoporosis at the femoral neck in HBV patients was lower than that at the lumbar spine. It might partly be explained by the reason that the turnover of cortical bone was lower than that of trabecular bone and the lumbar spine in chronic liver disease patients was mostly affected by cirrhosis.^8^ However, it was still controversial. Guggenbuhl P et al.^9^ reported lower BMD in the femoral neck compared the lumbar spine. While Valenti L et al.^10^ suggested an increased prevalence of osteoporosis at lumbar spine compared to femoral neck.

As we know, senile osteoporosis and postmenopausal osteoporosis are the main etiologies of primary osteoporosis. Our study showed that the osteoporosis incidence increased with age in HBV patients. Moreover, women was found with higher prevalence of osteoporosis than men in HBV patients and that prevalence increased significantly in the postmenopausal women. Our findings are consistent with previous study.^11^ Some studies reported that lower BMI showed lower BMD in chronic liver diseases.^12^ However, the association between BMI and BMD in HBV patients was not observed in our study.

The mechanisms for the association between HBV and osteoporosis are not clear. Chronic inflammation and decompensated liver or cirrhosis induced by HBV may be the potential mechanism. First, inflammatory cytokines (e.g., interleukin-1 (IL-1), and interleukin-6 (IL-6), tumor necrosis factor-alpha (TNFα)) associated with chronic HBV infection can increase receptor activator of nuclear factor kappa-B ligand (RANKL) to stimulate osteoclastogenesis and bone resorption.^13^ Moreover, TNFα was reported to inhibit the osteoblast differentiation and promote osteoblast apoptosis.^13^ The combined effects of these inflammatory cytokines can result in bone formation decreasing and bone resorption increasing, leading to reduced bone mineral density and then causing osteoporosis. Second, chronic HBV infection associated liver decompensation and cirrhosis can impair insulin-like growth factor 1(IGF-1) produced by liver, which inhibit osteoblast differentiation and proliferation promoted by IGF-1. In addition, previous study showed that hypogonadism observed in decompensated liver could increase osteoclast activity with reducing of estrogen and testosterone in blood levels.^14^ Third, metabolic acidosis in decompensated cirrhosis can also reduce bone mineral density through inducing calcium efflux from bone.^15^ Finally, decreasing of hydroxylation of vitamin D3 to D25 and blood levels of osteocalcin in advanced liver disease can accelerate bone loss and decrease bone formation.^16^

The measurement of BMD has been widely recommended in patients with chronic liver disease. Patients with cirrhosis were recommended with BMD assessment according to the societies of American and British gastroenterology.^17^,^18^ A recent report showed that chronic hepatitis B infection was associated with risk of hip fracture.^19^ Moreover, it was estimated that fracture risk would be increased twofold in cirrhosis regardless of aetiology^2^ and cumulatively by two fold or threefold for each decrease of one SD in BMD. In clinical practice, fracture risk could be prevented through the implementation of therapeutic measures by the systematic determination of BMD to limit the morbidity of patients with cirrhosis.^20^

### Limitations of the study

First, bone health associated with virus content was not determined in the present study. Second, lifestyle factors except smoking of osteoporosis were not fully ascertained in this study. Third, our analyses accounted only for baseline of BMD measurement but not use during follow-up.

## CONCLUSION

The present study demonstrated that chronic HBV infection was associated with low BMD and increased the risk of developing subsequent osteoporosis. Future studies are needed to confirm these findings, to determine the potential mechanisms and to evaluate the changes of bone mass during follow-up in these patients.

### Authors’ Contribution

**XZL** conceived, designed and did statistical analysis & editing of manuscript.

**ZFH, HW & CC** did data collection and manuscript writing.

**JW & SHY** did review and final approval of manuscript.
